# The Impact of Artificial Intelligence in the Odyssey of Rare Diseases

**DOI:** 10.3390/biomedicines11030887

**Published:** 2023-03-13

**Authors:** Anna Visibelli, Bianca Roncaglia, Ottavia Spiga, Annalisa Santucci

**Affiliations:** 1 Department of Biotechnology, Chemistry and Pharmacy, University of Siena, 53100 Siena, Italy; 2 Competence Center ARTES 4.0, 53100 Siena, Italy; 3 SienabioACTIVE—SbA, 53100 Siena, Italy

**Keywords:** rare disease, machine learning, artificial intelligence, precision medicine, data analysis

## Abstract

Emerging machine learning (ML) technologies have the potential to significantly improve the research and treatment of rare diseases, which constitute a vast set of diseases that affect a small proportion of the total population. Artificial Intelligence (AI) algorithms can help to quickly identify patterns and associations that would be difficult or impossible for human analysts to detect. Predictive modeling techniques, such as deep learning, have been used to forecast the progression of rare diseases, enabling the development of more targeted treatments. Moreover, AI has also shown promise in the field of drug development for rare diseases with the identification of subpopulations of patients who may be most likely to respond to a particular drug. This review aims to highlight the achievements of AI algorithms in the study of rare diseases in the past decade and advise researchers on which methods have proven to be most effective. The review will focus on specific rare diseases, as defined by a prevalence rate that does not exceed 1–9/100,000 on Orphanet, and will examine which AI methods have been most successful in their study. We believe this review can guide clinicians and researchers in the successful application of ML in rare diseases.

## 1. Introduction

The term rare diseases refers to a vast set of diseases that affect a small proportion of the total population; there are more than 7000 known disorders, and an estimated 250 rare new diseases are discovered annually [1]. In addition, diseases with a prevalence of <1 case per 50,000 population are defined as ultra-rare [2]. This definition is related to a prevalence threshold [3], and it differs depending on the jurisdiction. In Europe, the European Medicines Agency considers a prevalence of less than 5 in 10,000 people (less than 1 in 2000) [4], while in the United States, diseases affecting less than 200,000 people in the country were defined as rare by the Orphan Drug Act in 1983 [5]. In Japan, the Ministry of Health, Labor, and Welfare defines a threshold of fewer than 50,000 individuals in the country (equivalent to less than 1 in 2500 people) [6]. Therefore, an international definition of rare disease is lacking. Although individually they can be considered rare, they collectively afflict more than 500 million people worldwide [7]. Most of these disorders have characteristics that pose serious challenges for both researchers and public health professionals, especially for patients who face not only a loss in terms of health and quality of psychological and social well-being, but also financial burdens [8].

First, the process of diagnosing a rare disease is often long and exhausting. In 25% of patients, it takes between 5 and 30 years after disease onset to receive a correct diagnosis, which requires the participation of a competent and comprehensive clinical team [9]. A survey conducted from October 2019 through March 2020 by the National Organization for Rare Disorders [10] on 1108 individuals found that 50% of patients and caregivers attribute diagnostic delays to a lack of knowledge about the disease, while 42% believe that delays are caused by limited medical specialization. Many patients identified the problem of doctors not being able to link symptoms, particularly between different organ systems, in addition to the fact that waiting times to consult specialists are long and there would be a need for more tests. A diagnostic delay can have tremendous effects on the patient’s clinical picture, so prompt and accurate diagnosis are the starting point for being able to find therapeutic interventions and resources that can ensure a good clinical outcome [11].

What complicates the rare disease odyssey is that the diagnosis is never the end of the journey, since even from a prognostic and therapeutic point of view, there are huge gaps to be filled [12]. The difficulties encountered at the prognostic level are related to the lack of valid parameters and/or biomarkers, since the molecular pathophysiological mechanisms are still largely unknown. Moreover, the small number of patients does not allow statistically significant parameters to be derived [13]. Thus, the prognosis of patients may change depending on various genetic and environmental factors, but it is complicated to arrive at standards of care for treatment and rehabilitation because health research is necessarily conducted on a small scale and cannot be based on evidence or experience [14]. Conventionally, it takes 10 to 15 years to bring a drug to market, with an average R&D cost of $2.6 billion [15]. These two factors represent a bottleneck in the drug discovery pipeline for rare disorders, as research costs are high while revenues are low due to the small number of patients. This implies that the development of new drugs and treatments can be time-consuming and hindered by the lack of data and funding [16]. The heterogeneous patient populations, often unknown etiology and pathogenesis, the timing of disease progression, and the lack of exhaustive clinical studies make the search for specific drugs very difficult [17]. The key problems related to the development of drugs and therapies for rare diseases are [13]:

- only small cohorts of patients are interested in purchasing these drugs, making them so-called orphan drugs because they are not competitive for pharmaceutical companies.
- difficulties in treatment because most rare diseases are caused by genetic errors and/or have a degenerative nature.
- significant percentages of patients do not respond to available therapies due to partial or complete loss of response.

Therefore, rare diseases are often referred to as orphans as they fail to attract political, financial, and research interests, even though laws have been passed over the years to address this problem; the US Orphan Drug Act in 1983 and the European Union Regulation on Orphan Medicines in 2000 have rewarded innovation and focused on the value of healthcare for rare disease patients. Nevertheless, for most of them, there are no adequate therapeutic options. Over the years, specialized interdisciplinary centers for rare disorders have been established, where doctors and researchers can exchange opinions and ideas, creating networks of knowledge and experience that can help patients [9].

Possible innovative answers to biomedical and clinical challenges come from the world of information technology, and a striking example has been the fight against COVID-19. Since the beginning of the pandemic, artificial intelligence (AI) has played a crucial role in the battle against the virus, and several methods have been applied for various purposes [18]. Machine learning (ML) and deep learning (DL) models have been used for the early detection and diagnosis of COVID-19 by monitoring the demographic, clinical, and epidemiological characteristics of patients, and for developing diagnostic tools that can quickly analyze CT scans and X-rays to identify patterns indicative of the disease [19]. AI has also been used to predict patient vulnerability, in order to administer appropriate drugs and treatments [20], as well as being decisive in accelerating the discovery of potential vaccines. Similarly, AI has been an essential ally for public health policies in contact tracing, monitoring the spread of the virus, and creating predictive models that have helped to identify potential outbreaks. Thus, it is clear how AI is increasingly coming to the aid of physicians at every stage of disease management, to evaluate the efficacy of medical treatments or deeply investigate the correlation between patients and treatments according to their own molecular characteristics. The precision medicine approach is widely applied to the healthcare area, in particular to rare diseases with the creation of patient registries leveraging large amounts of data to discover potential links. It is a comprehensive and prospective approach to prevention, diagnosis, treatment, and monitoring, built on the genetic characteristics of the individual. Harmonizing databases and including registries are the major facilitators to understand the complexity of diseases, to conduct clinical trials, to improve the drug development process, and to assign the right treatment to the right individual after reliable patient stratification. AI is an ally that can integrate and analyze heterogeneous data (e.g., multi-omics data as well as images). However, first-generation AI systems, which rely on the development of algorithms for diagnosis and treatment that are trained on big data, are not always adequate to meet the needs of rare diseases. Data scarcity and sparsity characterize these disorders, due to fragmented knowledge and the limited number of data and specimens available [21]. Phenotype and disease severity as well as pharmacogenomic and pharmacokinetic factors are the elements on which successful diagnosis and treatment depend [13]. Diagnostic decision support systems (DDSS) already exist, i.e., expert systems that support doctors in facilitating the diagnostic process by incorporating medical knowledge. These systems have been proven effective and have improved clinical diagnosis by compiling lists of appropriate differential diagnoses for a given sample of tests [22,23]. For rare diseases, these systems need to be implemented. Networks and registries have been built to bring together data and expertise on rare diseases, making them free, accessible, and shareable worldwide. One example is Orphanet, which over the past 20 years has become the go-to source for information on rare diseases, facilitating access to information and means to identify potential patients, and contributing to the development and sharing of knowledge. Other available datasets are the Online Mendelian Inheritance in Man and Human Phenotype Ontology. The knowledge deposited in these databases is used by DDSSs built for rare disorders, some examples of which are FindZebra [24], PhenoTips [25], Rare Disease Discovery [26], and Ada DX [27].

**Figure 1:**
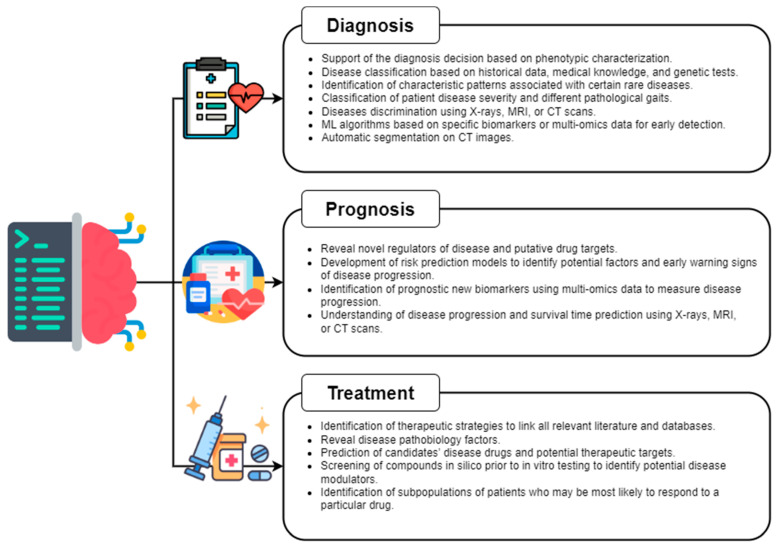
Examples of ML applications within diagnosis, prognosis, and treatment.

Second-generation AI systems were designed to fill the diagnostic, prognostic, and therapeutic gaps that must be overcome to achieve patient-centricity for patients with rare diseases [28] (Figure 1). These systems use a personalized closed-loop system designed to enhance end-organ function, overcome problems of tolerance or loss of efficacy, and improve patients’ responses to chronic drugs [13] in a precision medicine perspective.

So far, two scoping reviews [29,30] have been written on the use of ML and DL in rare diseases. The purpose of this review is to highlight the successes of AI algorithms in the study of rare diseases in the past decade and provide researchers with guidance on which methods have proven to be most effective. The review will focus on specific rare diseases, as defined by a prevalence rate that does not exceed 1–9/100,000 on Orphanet, and examine which AI methods have been most successful in their study.

## 2. Artificial Intelligence

The scientific field called AI [31] tries to develop robots that can mimic human perception and acquire the ability to solve problems for themselves. The fundamental subsets of AI are ML, which is based on the premise that computers can learn to execute certain jobs by gaining experience and improving their skills, and DL, which includes models of increasing complexity and abstraction. At the base of ML there are a series of different algorithms which, starting from primitive notions, learn to make a specific decision or to perform actions learned over time. Only a collection of data (training set) is given to the machine, which is iteratively evaluated to extract information, similarly to how humans learn.

Based on how the computer learns data and information, four distinct learning approaches can be identified: supervised learning [32], unsupervised learning [33], semi-supervised learning [34], and reinforcement learning [35]. Furthermore, the ML process consists of six components regardless of the algorithm adopted [36]. Data collection and pre-processing refer to the preparation of data, which is generally unstructured and sparse, in a format suitable for the algorithm’s input. The second step includes the standardization of the dataset, which helps to learn algorithms to avoid bias in the results. Later, feature selection can be applied to limit the number of input variables and reduce computational costs, thus improving efficiency. Even so, not all ML techniques are appropriate for all issues; rather, specific algorithms are better suited to a particular class of challenges. Any ML model’s ultimate goal is to learn from examples in a way that allows it to apply what it has learned to novel situations never encountered before. The model should then be trained on a subset of the total dataset, and its performance should then be measured against unknown data. Moreover, an ML tool may also do a wide range of tasks:

- Classification: two or more classes are created with the input data, and the learning system aims to produce a model capable of assigning a class to each input.
- Regression: conceptually similar to classification, with the difference that the output is continuous.
- Clustering: data are divided into groups about which there is no prior knowledge.

Decision trees, genetic and boosting algorithms, and metric techniques, such as the K-nearest neighbor algorithm (k-NN), Support Vector Machines (SVM), statistical approaches, Bayesian models (BM), Artificial Neural Networks (ANN), and Ensemble methods are all examples of ML techniques, which have a wide range of applications across various disciplines.

Numerous instances of multidisciplinary research in the particular context of experimental biology and biomedicine have shown the potential effectiveness of these methods. The most common AI techniques used include the DL subclass, which has opened the door to exploring tasks that would be difficult to address using shallow approaches [37]. Although it has a wide range of applicability, AI is still not a common technology employed by scientists. To be precise and effective, ML algorithms require large volumes of data, which is a challenging task in the biological field because digital information is scattered and not always accessible, making it difficult to make accurate predictions.

## 3. AI Application in Rare Diseases

To identify scientific articles that apply ML in the field of rare diseases, papers containing ML data on rare diseases were searched on Pubmed. The search strings we used include general terms related to AI (“artificial intelligence”, “machine learning”, “deep learning”) and diseases (“rare disease”, “ultra-rare disease”, “orphan disease”). Thus, we chose to include papers on diseases defined by a prevalence rate that does not exceed 1–9/100,000 on Orphanet. We included papers published between 1 January 2013, and 31 December 2022, and the studies identified in the search had to fulfill the following eligibility criteria: rare disease topic and use of at least one ML method with sufficient detail to extract the basic information analyzed in this review. Twenty-nine unique rare diseases were identified from the reviewed articles. After having selected relevant studies according to the eligibility criteria, we divided them based on the medical application, i.e., diagnosis, treatment, and prognosis. Then, the medical study, input data, and algorithm type and performances were assessed in detail for each study.

### 3.1. Diagnosis

Accurate diagnosis of rare diseases is an important task in patient triage, risk stratification, and targeted therapies. Rare disease symptoms often appear unfamiliar and atypical to a clinician due to their infrequency, and the likelihood that patients will not get an appropriate diagnosis and subsequent successful therapy is highest. The variability of rare diseases also makes it difficult to identify corresponding diseases in a timely manner due to the lack of clinical diagnostic procedures accessible.

A typical approach for the diagnosis of a rare disease includes a thorough medical history, physical examination, and genetic testing, which may identify specific mutations that are associated with the disease. Additionally, imaging studies such as X-rays, MRI, or CT scans may also be used. In this context, AI has the potential to play a significant but challenging role, through the development of ML algorithms that can analyze large amounts of data to identify patterns and markers that are characteristic of specific rare diseases. Moreover, AI-based diagnostic tools can also help to reduce the time and costs associated with diagnosing rare diseases by identifying potential diagnoses more quickly and accurately. Many ML techniques have been created to help in standardizing and sharing clinical and medical words through diverse medical resources, in order to improve inter-operability in the field of rare diseases. However, ML algorithms often require a significant number of training examples to achieve a good generalization performance, while the number of relevant clinical records in this field is bounded by the size of the population.

New strategies have been used to compensate for the lack of training data for rare disease diagnosis. For example, in [38], based on the requirement of providers to document associated phenotypic information to support a diagnosis, authors hypothesize that patients’ phenotypic data stored in electronic medical records can be used to speed up disease diagnosis. In this study, they suggested a collaborative filtering method enhanced with natural language processing and semantic techniques to help with phenotypic characterization-based rare disease identification. The preliminary results obtained demonstrated that the use of collaborative filtering with phenotypic information can stratify patients with relatively similar rare diseases. In [39], the phenotype-based Rare Disease Auxiliary Diagnosis system was developed, adopting both the traditional phenotypic similarity method and a new ML method to build four diagnostic models to support the diagnosis of rare diseases. Each model provides, with high diagnostic precision, a list of the top 10 candidate diseases as the prediction outcome. In another study [40] based on the fact that clinical symptoms in children with pulmonary diseases are frequently non-specific, authors developed and tested a questionnaire-based and data mining-supported tool, providing diagnostic support for selected pulmonary diseases. Eight different classifiers and an ensemble classifier were developed and trained to categorize any given new questionnaire and suggest a diagnosis. All questionnaires of patients suffering from cystic fibrosis, asthma, primary ciliary dyskinesia, acute bronchitis, and the healthy control group were correctly diagnosed by the fusion algorithm and exhibited good results in arriving at diagnostic suggestions. Moreover, due to the very nature of rare diseases, the lack of historical data poses a great challenge to ML-based approaches in accurately identifying rare diseases based on symptom descriptions. This work [41] used medical knowledge in automatically constructed knowledge graphs to develop a rare disease classification algorithm, delivering robust performance and outperforming a wide range of baselines.

More than one method has been applied to Huntington’s Disease (HD). This is a rare, inherited, neurodegenerative disorder that causes the progressive breakdown of nerve cells in the brain and leads to the loss of cognitive, behavioral, and physical abilities. It typically develops between the ages of 30 and 50, and the most visible symptom is chorea, which consists of involuntary movements of the upper and lower extremities, face, or body, and occurs in about 90% of patients. There is currently no cure for HD, but treatments are available to manage symptoms and improve quality of life. Reliable markers measuring disease progression in HD, before and after disease manifestation, may guide a therapy aimed at slowing or halting disease progression. ML methods have been widely used for gait assessment through the estimation of spatio-temporal parameters, demonstrating that the application of supervised classification methods is a valuable and promising approach to the automatic detection of disease stages in HD. In [42], Zhang et al. investigate the potential of classifying patient disease severity based on individual footstep pressure data using DL techniques. Using the Motor Subscale of the Unified HD Rating Scale as the gold standard, the experiments performed showed that use of VGG16 and similar modules can achieve high classification accuracy. The objective of the work described in [43] was instead to propose a validated SVM classifier that takes advantage of Hidden Markov Model-derived information for the classification of different pathological gaits. Specifically, the presented methodology allowed for proper discrimination against gait data from HD patients and healthy elderly controls using data from inertial measurement units placed at the shank and waist. Furthermore, alterations in oculomotor performance are among the first observable physical alterations during the pre-symptomatic stages of HD. In the pre-symptomatic and early symptomatic stages of HD, quantifiable assessments of oculomotor function have been investigated as potential markers of disease state and development. In [44], Miranda et al. reported the application of the SVM algorithm to oculomotor features pooled from a four-task psychophysical experiment. They were able to automatically distinguish control participants from pre-symptomatic HD participants and HD patients with high accuracy. Finally, quantitative electroencephalography (qEEG) may also provide a quantification method for possible sub-cortical dysfunction occurring before, or concomitant with, motor or cognitive disturbances observed in HD. In this pilot study [45], the authors constructed an automatic classifier, distinguishing healthy controls from HD gene carriers using qEEG. Derived qEEG features that correlated with clinically known markers represented new potential biomarkers of HD disease progression.

Starting from the assumption that bio-imaging technologies are increasingly impacting life sciences, and that sharing of image data is required to enable innovative future research, there are several rare disease studies that use images as input data. Parkinson’s disease (PD) and multiple system atrophy (MSA) are two neurodegenerative diseases that can have overlapping clinical manifestations. MSA is a progressive rare neurodegenerative disorder characterized by a combination of symptoms that affect both the autonomic nervous system and movement. This is caused by the progressive degeneration of neurons in several parts of the brain and spinal cord. The objective of the studies described in [46,47] were to assess the potential of SVM techniques to distinguish between PD and MSA patients at the single-patient level. Measures of cerebellar-brain network and cerebellar-striatal connectivity and subcortical edge-wise tractography data were used as predicting features in the articles respectively. Convolutional neural networks (CNN) were used in [48] to distinguish each representative parkinsonian disorder using a single midsagittal MRI. CNN enabled accurate discrimination among PD, progressive supranuclear palsy, MSA with predominant parkinsonian features, and normal status, although the dataset was limited.

Amyotrophic lateral sclerosis (ALS) is also a neurodegenerative rare disorder that affects nerve cells in the brain and spinal cord. The disease is progressive and leads to increasing disability, with patients eventually losing the ability to speak, swallow, and breathe. There is no known cure for ALS, and treatment options are focused on managing symptoms and prolonging survival. In [49], a deep CNN was developed for the classification of ALS patients and healthy individuals. Based on the recent insight that regulatory regions harbor the majority of disease-associated variants, authors employed a two-step approach: promoter regions that are likely associated with ALS have been identified, and individuals were classified based on their genotype in the selected genomic regions to identify potentially ALS-associated promoter regions. The application of a new advanced neuroimaging method, which delineates the profile of tissue properties along the corticospinal tract of patients with ALS using diffusion tensor imaging (DTI), was described in [50]. RF was used to assess the clinical utility of DTI in discriminating ALS from controls, with the potential to be of diagnostic utility in ALS. Finally, in [51], the authors utilized independent component analysis to derive brain networks based on resting-state functional magnetic resonance imaging and used those derived networks to build an ALS disease state classifier using SVM.

More generally, SVM methods have been widely and differently applied in the field of rare diseases. In this study, Palstrøm et al. [52] aimed to improve the diagnosis of amyloidosis by developing unbiased models based on proteomics data for the recognition of amyloid-containing biopsies, followed by accurate subtyping of amyloidosis. The authors demonstrated that using SVM on proteomics data can identify and classify patients with high accuracy. Hypophosphatasia is a rare genetic disease in which patients may have stress fractures, bone and joint pain, or premature tooth loss. In [53], the authors developed several ML algorithms based on specific biomarkers of this disease, determining the best way to diagnose this condition. SVM was the ML algorithm that provided the best predictive models in terms of classification. Nguyen, et al. [54] proposed a measuring instrument based on ML to quantitatively assess impairment levels while engaged in daily activity, for monitoring the progression of neurodegenerative conditions of Friedreich ataxia. Movement patterns during a simulated eating task were captured and kinematic biomarkers were extracted that were consistent with the frequently used clinical rating scales. SVM and other methods have been shown to accurately classify individuals with Friedreich ataxia and control subjects. The work in [55] aimed to assess the feasibility of a supervised ML algorithm for the assisted diagnosis of patients with clinically diagnosed progressive supranuclear palsy (PSP), a rare neurodegenerative disorder that shares similar clinical symptoms with PD. Morphological MRI of PD patients, PSP patients, and healthy control subjects was used as the input of a supervised ML algorithm based on the combination of PCA as a feature extraction technique and SVM as a classification algorithm. The authors in [56] characterized the 3D structure of the cortical bone in high-resolution micro-CT images to analyze the micro-structural properties of bone in cases of osteogenesis imperfecta (OI), a genetic disorder of connective tissues caused by an abnormality in the synthesis or processing of collagen. Numerous features computed from the image were used in an SVM model to classify between healthy and OI bone.

ANN and DL models have been shown to be highly effective in identifying and classifying diseases, and are becoming increasingly popular in the medical field as a tool for accurate and efficient diagnosis. In both [57,58], NN models were applied to eye photographs with the aim of identifying rare diseases. A hybrid learning-based neural network classifier (HLNNC) was implemented in [57] to identify mucormycosis disease by comparing images of patients with and without mucormycosis, a rare fungal infection caused by a group of molds. In [58], the discrimination ability of a deep CNN for ultrawide-field pseudocolor imaging and ultrawide-field autofluorescence was demonstrated for the detection of retinitis pigmentosa, a complex hereditary eye condition that causes cells in the light-sensitive retina to degenerate. Using the proposed model, retinitis pigmentosa was distinguished from healthy eyes with high sensitivity and specificity on ultrawide-field pseudocolor and ultrawide-field imaging. Automatic segmentation was instead implemented in [59,60]. In the first study, a deeply supervised 3D V-Net was used to automatically segment the arteriovenous malformations volume on CT images, demonstrating its clinical feasibility by validating the shape, positional accuracy, and dose coverage of the automatic volume. In the second study, a DL approach based on a holistically-nested network reliably segmented the lung across the breathing cycle to accurately analyze the lung and respiratory muscle movement in Duchenne muscular dystrophy. This is a severe form of childhood muscular dystrophy that affects 1 in 5000 boys, characterized by progressive muscle degeneration caused by alterations in a protein that helps to keep muscle cells intact. In [61], authors constructed an ANN diagnostic model capable of differentiating primary immune thrombocytopenic purpura (pITP) patients and established a potential pITP diagnosis platform. pITP is defined as isolated autoimmune thrombocytopenia with idiopathic low platelet count, normal bone marrow, and unexplained causes of thrombocytopenia. In a recent study described in [62], authors studied multiple osteochondromas, an autosomal dominant disease characterized by the formation of osteochondromas or exostoses. The aim of this study was to create an efficient system based on a switching neural networks approach to characterize multiple osteochondromas patients in three classes, according to the number of bone segments affected, the presence of skeletal deformities, and functional limitations. Finally, due to the urgent need for biomarkers for the early detection of neurodevelopmental spectrum disorders, in [63], authors applied a trained neural network, ConvNetACh, with heart rate variation data of Rett syndrome patients, capable of distinguishing them from subjects showing typical development.

Ensemble learning (EL) can help to improve the accuracy of rare disease diagnosis by combining the predictions of multiple models and leveraging the strengths of each individual model. This can be particularly useful in the context of rare diseases, where the number of cases is limited and the diagnostic criteria can be complex. Pulmonary arterial hypertension (PAH) is a rare but progressive cardiopulmonary disease that leads to heart failure and premature death. MicroRNAs are small, non-coding molecules of RNA, previously shown to be dysregulated in PAH, and contribute to the disease process in animal models. In [64], EL techniques were used to select miRNAs able to distinguish PAH and healthy controls. These circulating miRNAs and their target genes may provide insight into PAH pathogenesis and reveal novel regulators of disease and putative drug targets. Primary sclerosing cholangitis (PSC) is a rare, chronic, cholestatic liver disorder characterized by inflammation and fibrosis in the bile ducts, and it is known for its frequent concurrence with inflammatory bowel disease. Dysbiosis of the gut microbiota in PSC was reported in several studies, but the microbiological features of the salivary microbiota in PSC have not been established. In [65], Iwasawa et al. implemented a random forest (RF) algorithm able to distinguish the salivary microbial communities of PSC patients, ulcerative colitis patients, and healthy controls, indicating the potential of salivary microbiota as biomarkers for the non-invasive diagnosis of PSC. In [66], an ML method based on RF was developed to automatically detect the early deterioration of photoreceptor integrity caused by inherited retinal degenerative diseases. An application example is choroideremia, which is an X-linked chorioretinal dystrophy characterized by progressive degeneration of the choroid. This tool can be used for choroidal flow assessment in order to provide a more comprehensive description of disease progression. Finally, authors in [67] used RF methodology in patients with three groups of rare myopathic conditions, which includes any disease that affects the muscles that control voluntary movement, showing that the methodology was able to classify myotonic dystrophy type 1 and inflammatory myopathy. Table 1 summaries the described above ML methods applied for the diagnosis of rare diseases.

### 3.2. Prognosis

The prognosis includes information about the likely or expected evolution, duration, and outcome of the condition. In most cases, the possibility of a cure is also mentioned; however, most rare conditions are chronic and lifelong, so the goal is to manage the condition rather than cure it [6]. The difficulty in making a predictive prognosis not only affects the physical health of the patient, but also their mental health, leading to stress, anxiety, and depression [68]. 

AI can play a significant role in the prognosis of rare disorders by helping to fill in the gaps in data and experience [69]. By analyzing large amounts of data, such as electronic health records, genomic data, and imaging studies, ML algorithms can identify patterns and predict outcomes for individuals with rare diseases, providing valuable insights that can inform prognoses and guide decisions [70]. Additionally, AI can be used to develop new prognostic tools, such as risk prediction models, which can identify potential factors and early warning signs of disease progression, allowing for early intervention and potentially improving patient outcomes [71].

The commonly used AI approaches in the prognosis phase are supervised learning with EL, ANN, and SVM as the most widely used methods. Unsupervised methods, such as clustering, are used less frequently.

Two recent studies [72,73] used ML to identify new biomarkers that could be employed for prognostic purposes for adrenocortical carcinoma (ACC), a rare and aggressive cancer that arises from the cells of the outer layer of the adrenal gland. The prognosis for ACC is generally poor, with a 5-year survival rate of only about 10–20%, so early detection is crucial for improving the chances of survival, as well as identifying new markers. In [72], the authors applied a simple and unsupervised ML method called uniform manifold approximation and projection (UMAP) to mRNA expression data from the TCGA-ACC study, the largest multi-platform study of ACC. UMAP is a dimension reduction technique, and it found two distinct clusters that strongly correlated with patient prognosis. They then used an RF algorithm to identify the transcriptional differences between the two clusters, finding 100 genes that could serve as new biomarkers or novel targets for treatment. In [73], the authors performed a proteomic analysis of ACC at different stages and identified 7000 individual proteins. They selected 117 differentially expressed proteins (DEPs) using three feature selection algorithms (ReliefF, infoGain, and ANOVA) and conducted a survival analysis to assess the effect of the identified DEPs on patient survival. They were able to identify five new candidate protein biomarkers as prognostic factors, which can help in defining new therapeutic targets. Both studies highlight the importance of using ML with multi-omic data to better understand the biology of ACCs and to identify biomarkers for the disease.

The study of alkaptonuria is an example of how multiple ML techniques have been applied to an ultra-rare disease. Alkaptonuria (AKU) is an autosomal, recessive, and metabolic disorder caused by a defect in the enzyme homogentisic acid oxidase. As a result, homogentisic acid accumulates in the body and causes the formation of ochronotic pigments, and this can lead to various symptoms such as arthritis, amyloidosis, and kidney stones. Due to the rarity of the disease and the lack of a standardized method of assessment, studying AKU can be challenging. A recent study [74] has implemented a digital platform, ApreciseKUre, which is designed to collect, integrate, and analyze data for patients with AKU. The platform includes a wide range of data, including genetic, biochemical, histopathological, clinical, therapeutic resources, and quality of life (QoL) scores, which can be shared among researchers and clinicians to create a precision medicine ecosystem. The authors describe how ML applications were used to analyze and interpret the data in ApreciseKUre to achieve patient stratification, and tailor care and treatment to specific subgroups of patients. Two specific studies show the potential of ML in the context of AKU data. The first study [75] aimed to predict QoL scores based on patient’s clinical data using the XGBoost algorithm and a k-NN algorithm. The second study [76] aimed to compare different algorithms (K-means and hierarchical clustering) to explore phenotype-genotype relationships that were previously unknown in AKU. Both studies showed that ML successfully predicted clinical outcomes and QoL scores, and also identified new biomarkers and subgroups of patients with AKU. These studies highlight the need for the development of databases for rare diseases, helping to optimize the benefit-risk ratio, and improving overall patient outcomes.

ALS is another rare and very serious disease that has been studied with AI methods. In [77], the authors used pharmacometabolomics approaches and ML algorithms to identify metabolic changes in patients with ALS and the effects of two different treatments: riluzole and olesoxime. They applied multivariate statistical techniques such as partial least squares regression, orthogonal partial least squares discriminant analysis, and a novel algorithm called Biosigner. This algorithm, which is based on bootstrapping and different methods like RF and SVM, was found to have better predictive power than other approaches. The study found that certain lipids and amino acids were differentially expressed in the two treatment groups, and that these changes might be linked to changes in energy metabolism and glutamate metabolism, which are known to be important in ALS pathophysiology. In [78], Huang et al. present a novel non-parametric survival analysis method called GuanRank that aims to improve the reliability and robustness of survival predictions in clinical trials. This method is based on the Kaplan-Meier estimator and transforms the problem into a general regression problem that can be solved by ML regression algorithms such as Lasso regression, Gaussian process regression, and RF. The method was validated on the PRO-ACT database, a large de-identified dataset of patients in ALS clinical trials, and it demonstrated superior performance over the traditional survival models such as the Cox proportional hazard model. Gordon & Lerner [79] also used data from the PRO-ACT database to predict the state of ALS patients. They used RF, XGBoost, cumulative link models, ordinal decision trees, and cumulative probability trees as the prediction models and BM for knowledge representation. They found that ordinal classification models improved predictive performance and identified variables that were not previously known to be related to ALS, such as creatinine, CK, and phosphorus. In addition, data related to language and MRI images of ALS patients can be used to better understand the progression of the disease. Wang et al. [80] aimed to develop an automated assessment tool for speech impairment in ALS to improve the early detection and monitoring of bulbar dysfunction in ALS patients. They proposed the use of ML to detect abnormal speech patterns in ALS from both acoustic and articulatory samples and to help in the assessment of disease progression. The speech data is in the form of features extracted from speech recordings, which can be done using open-source algorithms such as openSMILE. Gradient boosting was used as the feature selection technique and SVM was used to predict intelligible speaking rate from speech acoustic and articulatory samples. In [81], the authors aimed to use DL to predict the survival time of ALS patients based on clinical characteristics and advanced MRI metrics. They collected high-resolution diffusion-weighted and T1-weighted images from 135 ALS patients at their first visit, and then monitored each patient’s survival time until death. Then, they used DL to create four different networks: one based on clinical data, one based on structural connectivity MRI data, one based on morphology MRI data, and one based on a combination of the three sources of information. The results showed that MRI data alone can provide valuable predictions of survival time and that combining clinical characteristics and MRI data into a DL approach can further improve predictions about a patient’s survival time. These studies on ALS highlight the importance of combining multiple sources of data such as clinical characteristics and MRI metrics to improve the accuracy of predictions.

As already seen for diagnosis, AI can be of great help in the prognostic phase of HD as well. Lauraitis et al. [82] proposed a hybrid model that uses artificial ANN and a Fuzzy Logic expert system (FLS) to predict, through finger-tapping tests, the deterioration of reaction state in individuals with neurological movement disorders such as hand tremors and non-voluntary movements. This model is composed of four sub-models (dataset formation, ANN prediction, FLS, and a decision module for determining the person’s condition) and was tested on a dataset of 3032 records from 20 test subjects. Results show that the feed-forward backpropagation neural network model achieved the best performance results. The authors plan to validate the proposed system using a larger dataset including data from PD and Alzheimer’s patients, as well as using more sophisticated finger-tapping features and comparing ANN results with those of SVM regression. In [83], they present a new approach that uses a combination of brain function and structure imaging data to identify whether a person with HD will receive a clinical diagnosis within 5 years, known as premanifest HD (preHD). The researchers used an SVM to classify individuals with preHD from controls. The input data were resting-state functional connectivity, subcortical gray matter volume, and cortical thickness. The SVM was trained using a linear kernel and a weighted cost function to account for class imbalances, and then the models were evaluated using leave-one-out validation and permutation testing. They also applied independent validation to test the generalizability of the findings. Asadi et al. [84] also wanted to predict the progression of a disease, i.e., cerebral arteriovenous malformations (cAVMs). They noticed that the lack of large observational studies on the long-term outcome of unruptured cAVMs has made it difficult to determine the best course of action. cAVMs are rare, abnormal connections between the arteries and veins in the brain that typically form before birth. They can vary in size and location, and may cause a rupture, leading to hemorrhage and reduced blood flow to the brain. Since cAVMs can present symptoms at any age, the goal is to identify factors that can be used to predict hemorrhagic risk and to develop a risk stratification model that can be used to guide treatment decisions. They used ANN and SVM to predict the outcome of cAVMs post-endovascular treatment with relatively high accuracy and precision. The ANN was found to be the strongest predictor of fatal outcome, with the presence or absence of nidal fistulae having the greatest predictive power. The study also found out that the classical regression model had mediocre accuracy in predicting the outcome of mortality, with the type of treatment-related complication being the most important predictor. In [85], the authors developed an ML algorithm based on DTI to predict the clinical severity of PSP. The algorithm was trained on data from a cohort of PSP patients and was found to be accurate in predicting the severity of the disease as measured by various clinical scales. Moreover, the algorithm identified regions of the brain related to motor function, such as the thalamus, and regions related to psychomotor interactions, such as the parahippocampus gyrus, that are associated with the severity of the disease.

Other examples of where SVMs have been successfully applied include the works of Zhutovsky et al. [86] and An et al. [87]. In [86], they wanted to determine the prognostic accuracy of clinical and structural MRI data of patients with a behavioral variant of frontotemporal dementia (bvFTD) presenting late-onset behavioral changes. This disorder presents with behavioral and cognitive symptoms that overlap with other neurological and psychiatric disorders, so the authors suggest that predictive biomarkers could facilitate early detection. They used data from 73 patients, divided into three groups based on 2-year follow-up diagnosis: probable/definite bvFTD, neurological, and psychiatric. They then used SVM classifiers to perform classification tasks and evaluated performance using cross-validation. They found that the combination of clinical and voxel-wise whole brain data showed the best performance overall, and concluded that the results show the potential for automated early confirmation of bvFTD using ML analysis of clinical and neuroimaging data in a diverse and clinically relevant sample of patients. In [87], the authors used the SVM model to study mutations that cause Diamond-Blackfan anemia (DBA), a rare hereditary disorder characterized by failure of erythropoiesis. They first conducted a comprehensive study on the structural basis of human RPS19 mutations that occur in DBA, based on its 3D structures, and then used this knowledge to train an SVM model to predict the pathogenicity of all possible missense mutations of RPS19. They used 29 DBA mutations (positive samples) and 30 neutral ones (negative samples) as training data, and extracted 8 features to be used for each mutation, such as interaction with rRNA, structural stability, and conservation. After five-fold cross-validation, the best hyperparameters were identified and the SVM model was able to predict 26 of the 29 DBA mutations correctly, with a significantly reduced false-positive rate compared to other prediction tools.

As mentioned, the most widely used type of AI algorithms for the prognosis of rare diseases are EL algorithms. Here, we give some examples of where these have been successfully applied. In [88], they used a bootstrap aggregation (bagging) ensemble technique with a reduced-error pruning regression tree as the underlying classifier to predict the energy expenditure (EE) of children with DMD. Existing ML algorithms developed for healthy populations do not accurately estimate EE for children with DMD, so they develop a custom ML model specifically for this population. Bagging is an ensemble meta-algorithm that improves the stability and accuracy of statistical regression obtained by a regression tree. They demonstrate that this technique has proven to be superior to other models such as multilayer perceptron, SVM, linear regression, naive Bayes, and reduced-error pruning regression tree. Another example of boosting algorithm application is [89], where the authors present a novel ML model named the PSC risk estimate tool (PREsTo) that predicts the 5-year risk of hepatic decompensation in patients with PSC. Due to the rarity of the disease, improved biomarkers are necessary to risk-stratify patients in clinical trials and serve as surrogate endpoints. The PREsTo model used gradient boosting machines, a step-wise method to create an ensemble of weak prediction models, typically decision trees. Each decision tree may have different variables, and the ones with the strongest predictive power are used more often and earlier in the model-building process. The study reported that the model performed well compared to the existing prognostic markers, had an excellent performance when applied to a later point in the disease course, and had good performance among various PSC subgroups. The advantages of this model are that it uses readily available clinical data, and it is non-invasive, inexpensive, and accurate. In [90], Robinson et al. used immune cell frequency profiles, clinical, and serological data from patients with juvenile-onset systemic lupus erythematosus (jSLE) to identify predictive disease outcome signatures using RF and sparse partial least squares-discriminant analysis (sPLS-DA).

BRF was used to overcome difficulties in obtaining validation datasets because it does not overfit to training data, and it was used to further define and validate the pathological immune cell profile of the disease. sPLS-DA was used as a secondary validation method to rank and validate the immunological variables by their distribution in patients with jSLE and healthy controls. The analyses identified 8 immune cell subtypes that were consistently correlated with jSLE patients, compared with healthy controls. Lastly, in the works of Chou & Ghimire [91,92], they applied RF algorithms to identify prognostic factors in pediatric myocarditis patients. In their first study [91], they used an RF algorithm on 500 factors from a publicly available pediatric hospitalization database (Kids’ Inpatient Database) to identify mortality risk factors, and validated these factors using linear and binomial regression models. They also used negative binomial regression models to study the association between the length of hospitalization and risk factors. The goal of the second study [92] was to develop a model to predict in-hospital mortality among patients hospitalized for pediatric myocarditis, since traditional logistic regression models have low sensitivity. A total of 14 variables were included in model development and an RF algorithm was applied because of the nature of the predictors, which are all two-level categorical variables. Based on the importance scores of the risk factors, the top 5 variables were selected as MV, ECMO use, cardiac arrest, ventricular fibrillation, and AKI. Table 2 summaries the described above ML methods applied for the prognosis of rare diseases.

### 3.3. Treatment

There is an urgent need to identify novel treatment options for rare diseases, which is a difficult challenge due to the lack of essential data including drug molecules, genes, and protein structure information. The speed at which new biomedical knowledge is being discovered makes it particularly challenging to connect disease mechanisms to drug action. Almost 95% of rare diseases do not have FDA-approved drug treatment and the increasing number of rare diagnoses puts pressure on scientists and clinicians to characterize these conditions and match patients with appropriate treatments [93]. As biomedical discoveries continue to generate big amounts of data, an opportunity emerges for AI to help in translating biomedical knowledge into a format that can be used to identify therapeutic strategies for patients. Recently, The Hugh Kaul Precision Medicine Institute created mediKanren [94], an AI platform based on knowledge graphs that uses the mechanistic insight of genetic disorders to identify therapeutic strategies, enabling an efficient way to link all relevant literature and databases. The method was tested by analyzing genetic data and publications of two rare disorders related to missense variants in the TMLHE and RHOBTB2 genes, revealing molecular mechanisms and pathways which have provided new therapeutic targets.

Currently, AI methods for treatment belong mostly to supervised learning, which uses labeled datasets to train algorithms able to classify or predict outcomes accurately. In [95], Bakkar et al. implemented the IBM Watson^®^ [96] method to screen RNA-binding proteins (RBPs) in the genome and identify additional RBPs involved in ALS. Numerous RBPs have been shown to be altered in ALS, making them a contributing factor in disease pathobiology. IBM Watson extracts domain-specific text features from published literature to identify new connections between entities of interest. From these annotated documents, Watson created a semantic model of the set of RBPs with known mutations that cause ALS, and then applied that model to a candidate set of all other RBPs to cluster all the candidates by similarity to the known set using a graph diffusion algorithm. Gated Recurrent Unit Cooperation-Attention-Network (GCAN) was used in [97] to predict drugs for rare diseases, with particular attention to Gaucher disease, a rare metabolic disorder in which deficiency of the enzyme glucocerebrosidase results in the accumulation of toxic quantities of certain lipids. Two heterogeneous networks were built for information enhancement; one network contains the father nodes of the rare disease, while the other network contains information on the son nodes. A biased random walk approach was used to collect data from the father and son nodes, where nodes were linked in a hierarchical relationship with two hop distances. The effectiveness of two Gaucher disease drugs predicted by GCAN has been established. In [98], authors showed interest in sialidosis, an ultra-rare lysosomal storage disease characterized by an excessive accumulation of glycoprotein-derived oligosaccharides. J. Klein et al. applied the so-called Assay Central software [99] to build Bayesian ML models to screen compounds in silico before in vitro testing. This approach has been applied to identify new compounds that can act as a potential disease modulator in the treatment of sialidosis. In [100], the authors used an RF classifier for the prediction of cell-penetrating peptides, which can facilitate the intracellular delivery of large therapeutically-relevant molecules. The goal was to deliver phosphorodiamidate morpholino oligonucleotides, a type of antisense therapy recently approved by the FDA for the treatment of DMD. Multi-output regression ML methodologies were implemented in [101] to predict the potential effect of external proteins on the signaling circuits that trigger Fanconi anemia-related cell functionalities. This rare condition causes genomic instability and a range of clinical features, including developmental abnormalities in major organ systems and a high predisposition to cancer [102]. Thanks to these models, over 20 potential therapeutic targets were detected. In the last study [103], Spiga et al. developed an RF model that performs a prediction of the QoL scores based on data deposited in ApreciseKUre. Predicted QoL scores were then correlated with the drugs taken by AKU patients, revealing that drugs typically used to treat AKU patients were effective in reducing pain, but some common drugs not related to specific AKU symptoms also showed a correlation with some QoL scores. Table 3 summaries the described above ML methods applied for the treatment of rare diseases.

## 4. Discussion

In this review, we studied the scientific literature of the last decade to understand which AI methods are most widely used in the field of rare and ultra-rare diseases, revealing that the most applied algorithms are SVM, RF, and ANN. One of the reasons these methods are so popular is their ability to handle complex, high-dimensional data, and indeed images were found to be the most widely used type of input. Medical imaging techniques, such as PET, CET, MRI, and ultrasound, produce large amounts of this type of data, which can be standardized and therefore easily processed by AI methods. Moreover, these methods can also learn from small datasets, as is often the case in rare diseases due to their low prevalence, and are able to identify important features, which can help researchers better understand the mechanisms underlying the disease and potentially identify new targets for treatment. Another point to highlight is that most studies have focused on using AI methods for the diagnosis and prognosis of rare diseases, which is a typical application of classification and prediction, and this is also understandable given the challenges associated with identifying and predicting rare disorders. However, it is worth noting that ML could also play a significant role in improving the treatment, accelerating drug development by identifying potential drug candidates, predicting their efficacy and optimizing their dosages, as well as identifying drugs already on the market and repurposing them for other diseases.

However, the application of ML models can also present various challenges, starting with the difficulty of applying them to unstructured and poorly standardized textual data, such as medical records. This is because the presence of label-noise and sparsity can lead to model overfitting, meaning that the method has high prediction accuracy on training data and low prediction accuracy on new evaluation data. In addition, the aforementioned most widely used methods focus on classification tasks while few algorithms are applied to the study of the biological mechanisms of these diseases. This is probably due to the complexity of the systems biology that are these disorders, which sometimes have molecular bases that are still unknown, or a pathological picture that is unclear and overlaps with that of other diseases. This implies that there is a scarcity of already developed and validated methods that can tolerate these constraints, but there is also a high demand for AI approaches from the biomedical world. It is therefore essential to intentionally develop methods and analytic workflows that can address these challenges, and it is up to the developers of such methods to ensure that the resulting models are reliable. Thus, to increase confidence in the end user (i.e., clinicians and researchers), explanations of the behavior of the developed algorithms must be provided, and robust error analysis must be conducted.

Another issue that needs to be addressed is that only few studies have validated their models on external datasets, thus assessing their potential in clinical practice. External validation studies can help assess the generalizability and reliability of the algorithm and determine its potential use in clinical settings, but unfortunately only a few of the reported studies have conducted this type of validation. This is partly due to the difficulty in this field of obtaining large and diverse datasets and the lack of standardized methods for data collection and analysis, but without adequate validation, there is a risk that the algorithms will produce unreliable or inaccurate results when applied to new datasets or patient cohorts. Solving this problem requires collaboration among researchers, clinicians, and data scientists to develop standardized methods, as well as sharing of data and algorithms to facilitate external validation studies.

## 5. Conclusions

ML methods have shown promise in the identification and diagnosis of rare diseases. With the vast amounts of data now available through electronic health records and heterogeneous databases, AI algorithms can help quickly identify patterns and associations that would be difficult or impossible for human analysts to detect. For example, researchers use ML algorithms to analyze patient data and identify characteristic patterns associated with certain rare diseases. By doing so, these tools can help to narrow down the list of possible diagnoses, making it more likely that patients will receive a correct diagnosis in a timely manner.

Predictive modeling techniques, such as DL, have been used to forecast the progression of rare diseases, allowing for earlier interventions and better treatment planning. This could potentially lead to a more accurate classification of rare diseases and enable the development of more targeted treatments. From a precision medicine perspective, by identifying biomarkers associated with a particular rare disease, AI algorithms can help to develop personalized treatment plans, helping to improve patient outcomes and reduce the risk of side effects.

AI has also shown promise in the field of drug development for rare diseases. AI algorithms can be used to analyze patient data and identify subpopulations of patients who may be most likely to respond to a particular drug. This can help to make clinical trials more efficient and increase the chances of a drug being approved for use in patients with rare disorders. Despite the potential benefits of ML, there are still challenges that must be overcome to fully realize its potential in the field of rare diseases. These include a lack of large, well-annotated datasets, and the need for interpretable models that can be easily understood and trusted by clinicians. Rare diseases usually present an unusually big data regime, which is characterized by huge omics data but a limited number of patients. The rarity of orphan patients, despite the presence of registries, still has a large impact on ML analyses, and thus open data can contribute significantly to support the modeling attempt. In the future, patient registries and open data need to be integrated, translating the largest amounts of data available into potential connections. Finally, a current limitation is the lack of interpretability, which makes it difficult for clinicians and researchers to understand algorithm outputs. In fact, explainability is one of the most debated topics for the application of AI in healthcare. While AI-based systems have been shown to outperform humans in certain analytical tasks, the lack of explainability continues to attract criticism. In this context, explainable AI approaches are the new frontier of ML applications in healthcare, in order to ensure the understanding, by both clinicians and patients, of the “mental process” followed by the artificial brain to reach a certain decision.

In conclusion, the application of ML techniques can greatly assist rare disease research and treatment, but to use it effectively, it needs to be implemented under the right ethical principles, avoid biases, and also be transparent for the patient. To fully tap into its potential, AI needs to be validated through clinical trials and real-world evidence. Furthermore, it needs to be accompanied by regulatory frameworks that ensure the safety and reliability of AI-based medical devices and diagnostic tools. More work is needed to overcome data-related challenges, ensure fair and trustworthy models, and help translate the research findings into practical applications that can benefit patients and their families.

## Figures and Tables

**Table 1:** Summary of studies investigating ML methods applied for the diagnosis of rare diseases.

Disease	Methods	Data Type	Sample Size	Model Performance	References
Huntington disease	ANN	footstep pressure data	180	ACC: 89%	[42]
	SVM	gait data	15 (post stroke patients), 17 (HD patients), 10 (Controls)	ACC: 90.5%	[43]
	SVM	eye tracking data	22 (Controls), 14 (pre-HD patients), 14 (HD patients)	ACC: 73.47%, TPR: 74.31%, TNR: 72.64% pre-HD vs. Controls ACC: 81.84%, TPR: 76.19%, TNR: 87.48% HD vs. Controls ACC: 83.54%, TPR: 92.62%, TNR: 74.45 % pre-HD vs. HD	[44]
	BM	quantitative electroencephalography data	26 (preHD patients), 25 (Controls)	TNR: 83%, TPR: 83%, ACC: 83%.	[45]
Multiple system atrophy	SVM	MRI data	30 (MSA patients), 62 (PD patients), 59 (Controls)	ACC: 77.17%	[46]
	SVM	MRI data	31 MSA patients, 65 PD patients, 54 (Controls)	ACC: 78%	[47]
	ANN	MRI data	125 (PD patients), 98 (PSP patients), 54 (MSA-P patients), 142 (Controls)	PD ACC: 96.8%, AUC: 0.995 PSP ACC: 93.7%, AUC: 0.982 MSA-P ACC: 95.2%, AUC: 0.990 Controls ACC: 98.4%, AUC: 1.000	[48]
Amyotrophic lateral sclerosis	ANN	whole-genome data	4511 (ALS patients), 7397 (Controls)	ACC: 77%	[49]
	RF	brain MRI data	24 (ALS patients), 24 (Controls)	ACC: 80%	[50]
	SVM	fMRI data	32 (ALS patients), 31 (Controls)	ACC: 71%	[51]
Amyloidosis	SVM	proteomics data	75 (amyloid positive), 78 (Controls)	ACC: 96–99%	[52]
Hypophosphatasia	SVM	clinical data	23 (HPP patients), 22 (Controls)	ACC: 90%, TPR: 87%, TNR: 93%, AUC: 0.936	[53]
Friedreich ataxia	SVM	kinematic data	30 (Friedreich patients), 14 (Controls)	ACC: 91%, TPR: 90%, TNR: 93%, AUC: 0.91	[54]
Progressive supranuclear palsy	SVM	MRI data	28 (PSP patients), 28 (PD patients), 28 (Controls)	ACC: 85.8%, TPR: 86%, TNR: 86% PD vs. Controls ACC: 89.1%, TPR: 89.5%, TNR: 89.1% PSP vs. Controls PSP vs. PD 84.7 87.5 83.8	[55]
Osteogenesis imperfecta	SVM	μCT images	21 (specimens of 13 OI patients), 19 (specimens from 15 Controls)	AUC: 96%	[56]
Mucormycosis disease	ANN	eye photographs	Not clear	ACC: 99.5%	[57]
Retinitis pigmentosa	ANN	ultrawide-field images	150 (RP patients), 223 (Controls)	AUC: 0.998, TPR: 99.3%, TNR: 99.1% of the ultrawide-field pseudocolor group AUC: 1.000, TPR: 100%, TNR: 99.5% of the ultrawide-field autofluorescence	[58]
Cerebral arteriovenous malformation	ANN	Brain CT images	80	DSC: 85.2% TPR: 88% TNR: 99%	[59]
Duchenne muscular dystrophy	ANN	cine MRI data	15 (Duchenne patients), 16 (Controls)	DSC: 97.2 for the sagittal view DSC: 96.1 for the axial view DSC: 96.6 for the coronal view	[60]
Immune thrombocytopenic purpura	ANN	proteomics data	64 (pITP patients), 70 (sITP patients), 82 (Controls)	TPR: 87.5%, TNR 69.7%, ACC: 75.0%	[61]
Multiple osteochondromas	ANN	clinical data	96 (class I), 137 (class II), 56 (class III)	Class I ACC: 94% Class II ACC: 80% Class III ACC: 79%	[62]
Rett syndrome	ANN	heart rate variation data	35 (Rett patients), 40 (Controls)	ACC: 88%	[63]
Pulmonary arterial hypertension	EL	microRNA expression data	64 (PAH patients), 43 (Controls)	TPR: 91%, TNR: 64%, ACC: 81%, AUC: 0.85	[64]
Primary sclerosing cholangitis	RF	bacterial 16S rRNA gene sequence data	24 (PSC patients), 16 (UC patients), 24 (Controls)	PSC AUC: 0.7423 UC AUC: 0.8756	[65]
Choroideremia	RF	OCT/OCTA data	16 (eyes with choroideremia), 5 (Controls eyes)	J: 0.876 ± 0.066	[66]
Dermatomyositis	RF	sonographic muscle images	11 (IBM patients), 19 (DM1 patients), 21 (PM-DM patients)	ACC: 78.4%	[67]

Performance Metrics Abbreviations: true positive rate (TPR), true negative rate (TNR), positive predictive value (PPV), negative predictive value (NPV), accuracy (ACC), mean reciprocal rank (MRR), Relative Absolute Error (RAE), Mean Squared Error (MSE), Root Mean Squared Error (RMSE), Concordance index (C-index), area under the curve (AUC), Coefficient of determination (R^2^), Confidence interval (CI), Sørensen–Dice coefficient (DSC), Jaccard similarity index (J).

**Table 2:** Summary of studies investigating ML methods applied for the prognosis of rare diseases.

Disease	Methods	Data Type	Sample Size	Model Performance	References
Adrenocortical carcinoma	clustering, RF	mRNA expression data	79	ACC: 96%	[72]
	DT	differentially expressed proteins	117		[73]
Alkaptonuria	XGBoost, k-NN	genetic, biochemical, histopathological, clinical, therapeutic resources, and QoL scores	129	RAE: 0.25, R2: 0.94	[75]
	custering		112		[76]
Amyotrophic lateral sclerosis	RF, SVM	metabolomics data	38 (treated), 36 (placebo)	TPR: 71.4%, TNR: 71.4%, PPV: 71.4%, NPV: 70.0%	[77]
	LASSO, RF	a subset of the PRO-ACT dataset (survival and clinical data)	6565	C-index: 0.7355	[78]
	RF, XGBoost, BM, DT	a subset of the PRO-ACT dataset	3772	ACC: 71–84.7%	[79]
	GBoost, SVM	speech acoustic and articulatory data	1832	R2: 0.712, RMSE: 37.562	[80]
	ANN	Clinical and MRI data	135	ACC: 84.4%.	[81]
Huntington disease	ANN, FLS	finger-tapping tests data	3032	R2: 0.98, MSE: 0.08	[82]
	SVM, EL	Neuroimaging data	19 (preHD), 21(Controls)	F1: 74%	[83]
Cerebral arteriovenous malformation	ANN, SVM, Log Reg	clinical and imaging data	199	ACC: 97.5%	[84]
Progressive supranuclear palsy	LASSO, Lin Reg	Imaging data	53	R2: 0.892	[85]
Behavioral variant of frontotemporal dementia	SVM	clinical and structural MRI data	73	ACC: 72–82%, TPR: 67–79%, TNR; 77–88%, AUC: 0.80–0.9	[86]
Diamond-Blackfan anemia	SVM	structural data of missense mutation	29 (positive samples), 30 (negative samples)	ACC: 95%, TPR: 90%, TNR; 98% F1: 94%	[87]
Duchenne muscular dystrophy	EL	inertial sensor (accelerometer) data	7	RMSE: 0.017	[88]
Primary sclerosing cholangitis	GBoost	clinical and laboratory data	509	C-index: 0.90	[89]
Juvenile-onset systemic lupus erythematosus	RF	Immunophenotyping data	67 (jSLE), 39 (Controls)	ACC: 86·8%	[90]
Pediatric myocarditis	RF	Diagnoses/procedures data from the Kids’ Inpatient Database	7241	CI: 95%	[91]
	RF, Log Reg		4144	TPR: 89.9% TNR: 85.8%, ACC: 87.9%	[92]

**Table 3:** Summary of studies investigating ML methods applied for the treatment of rare diseases.

Disease	Methods	Data Type	Sample Size	Model Performance	References
Amyotrophic lateral sclerosis	IBM Watson^®^	RNA-binding proteins	1478	AUC: 0.935	[95]
Multiple diseases, exp. Gaucher disease	ANN	disease, gene, and drug data	7000	Hits@10: 0.454, MRR 0.231	[97]
Sialidosis	BM	disease targets and accessible bioactivity data	57	AUC: 0.737, PPV: 34.5%, TPR: 91%, TNR: 58.7%, F1: 50%	[98]
Duchenne muscular dystrophy	RF	PMO activity data	64	ACC:72%, PPV:75%, TPR:69%	[100]
Fanconi anemia	EL	gene expression data	>11,000	R2: 0.62–0.97	[101]
Alkaptonuria	RF	genetic, biochemical, histopathological, clinical, therapeutic resources, and QoL scores	129	ACC: 70%	[103]

## Data Availability

No new data were created or analyzed in this study. Data sharing is not applicable to this article.
